# Tubeimoside I-induced lung cancer cell death and the underlying crosstalk between lysosomes and mitochondria

**DOI:** 10.1038/s41419-020-02915-x

**Published:** 2020-08-26

**Authors:** Kun Wang, Yujuan Zhan, Bonan Chen, Yuhua Lu, Ting Yin, Shikun Zhou, Weibin Zhang, Xiaodong Liu, Biaoyan Du, Xianli Wei, Jianyong Xiao

**Affiliations:** 1 Research Center of Integrative Medicine, School of Basic Medical Sciences, Guangzhou University of Chinese Medicine, 510006 Guangzhou, China; 2 Department of Pathology, Guangzhou University of Chinese Medicine, 510006 Guangzhou, China; 3 Department of Biochemistry, Guangzhou University of Chinese Medicine, 510006 Guangzhou, China; 4 Department of Anaesthesia and Intensive Care, The Chinese University of Hong Kong, 999077 Hong Kong, SAR China; 5 Department of Medical Instruments, Guangdong Food and Drug Vocational College, 510520 Guangzhou, China

**Keywords:** Lung cancer, Drug discovery

## Abstract

Cancer cells have developed chemoresistance and have improved their survival through the upregulation of autophagic mechanisms that protect mitochondrial function. Here, we report that the traditional Chinese anticancer agent tubeimoside I (Tub), which is a potent inhibitor of autophagy, can promote mitochondria-associated apoptosis in lung cancer cells. We found that Tub disrupted both mitochondrial and lysosomal pathways. One of its mechanisms was the induction of DRP1-mediated mitochondrial fragmentation. Another mechanism was the blocking of late-stage autophagic flux via impairment of lysosomal acidification through V-ATPase inhibition; this blocks the removal of dysfunctional mitochondria and results in reactive oxygen species (ROS) accumulation. Excessive ROS accumulation causes damage to lysosomal membranes and increases lysosomal membrane permeability, which leads to the leakage of cathepsin B. Finally, cathepsin B upregulates Bax-mediated mitochondrial outer membrane permeability and, subsequently, cytosolic cytochrome C-mediated caspase-dependent apoptosis. Thus, the cancer cell killing effect of Tub is enhanced through the formation of a positive feedback loop. The killing effect of Tub on lung cancer cells was verified in xenografted mice. In summary, Tub exerts a dual anticancer effect that involves the disruption of mitochondrial and lysosomal pathways and their interaction and, thereby, has a specific and enhanced killing effect on lung cancer cells.

## Introduction

Lung cancer is one of the most malignant cancers and is associated with the highest cancer-related fatality rate; its incidence rate in China has been on the rise, with the increase in the smoking population^1^. There has been considerable progress in the research on and the development of inhibitors targeting oncogenes, especially EGFR, and antibodies against PD-1, which is a cell surface receptor that belongs to the immunoglobulin superfamily and is expressed on T cells and pro-B cells^2^. Accordingly, a series of small-molecule drugs have been approved by the Food and Drug Administration; however, these novel drugs are not affordable for a large portion of patients in China^2^. Consequently, patients with lung cancer still have a poor prognosis^3^.

Resection combined with chemotherapy is the most commonly used strategy for cancer treatment^4^. First-line chemotherapeutic drugs, such as cisplatin and paclitaxel, were initially effective for the treatment of lung cancer cells, but their killing effect has reduced with time as cancer cells have developed various mechanisms for survival^5^. Only increasing the dosage of the drugs as a means of overcoming cancer cell chemoresistance, however, will aggravate the side effects and worsen the quality of life of patients. Therefore, it is important to investigate other methods by which the chemoresistance of cancer cells can be tackled. Chemotherapy drugs typically induce mitochondrial damage and mitochondria-dependent apoptosis, but the target cells may survive through the activation of autophagy pathways^6^. Autophagy is a self-eating process whereby cells recycle their wastes to maintain homeostasis. In the process of autophagy, damaged organelles are encapsulated by autophagosomes, which in turn fuse with lysosomes to form autolysosomes, within which the cargo is degraded by hydrolases^7^. Based on this information, it can be hypothesized that blocking the autophagy pathway may promote mitochondria-dependent apoptosis in cancer cells.

We screened the UNPD natural products library published by Dr. Gu^8^ and discovered the anticancer properties of Tubeimoside I (Tub), which is a compound isolated from Chinese medicinal plants of the Fritillaria genus^9^. On the one hand, Tub induced mitochondrial damage, and on the other hand, it simultaneously inhibited the autophagy flux. Thus, Tub may be a novel potential candidate for lung cancer chemotherapy. Although plants of the genus *Fritillaria* are commonly used to treat tumors in traditional Chinese medicine, the major components and the anticancer mechanisms remain poorly studied. Therefore, in the present study, we have investigated the anticancer effect of Tub under in vitro and in vivo conditions, in order to specifically understand the associated lysosomal and mitochondrial mechanisms and their interactions in relation to Tub-induced apoptosis in lung cancer cells.

## Results

### Tub induced mitochondrial fragmentation via dephosphorylation of DRP1 at serine 637

**Fig. 1:**
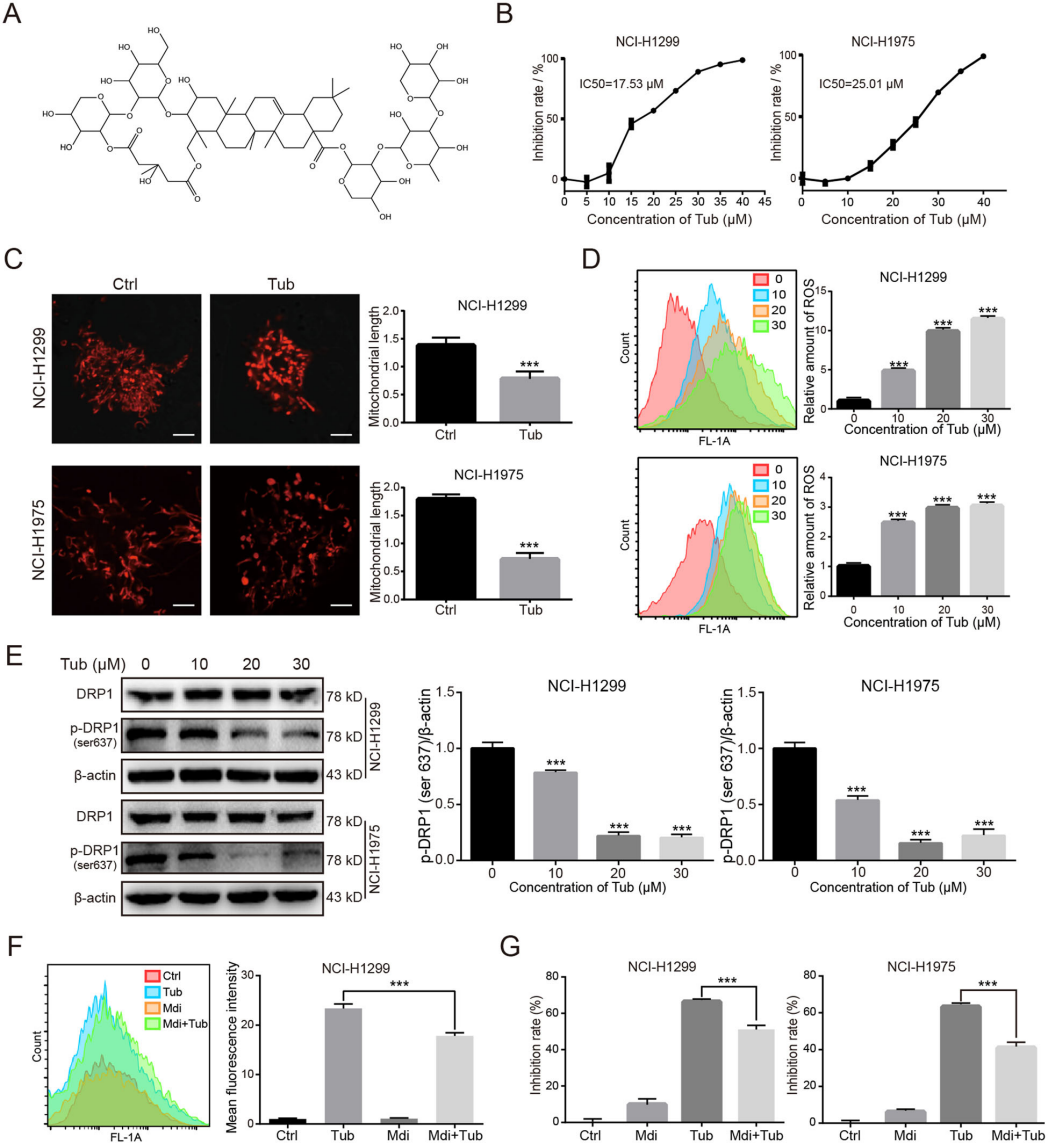
Tub induced mitochondrial fragmentation via downregulation of the phosphorylation of DRP1 at serine 637. **a** Chemical structure of Tubeimoside I. **b** Cells of the lung cancer cell lines NCI-H1299 and NCI-H1975 were treated with Tub at the indicated concentration gradient for 24 h. Cell viability was determined with the CCK8 assay, and IC50 was calculated with the help of the GraphPad 6.0 software. The inhibition of cell viability increased with increase in the Tub dose. **c** Tub (20 μM) induced the fragmentation of mitochondria after treatment for 24 h (mitochondria were labeled with MitoTracker Red). The length of mitochondria was quantified by the Image J software (scale bar = 5 µm). **d** Tub induced an increase in the intracellular ROS level. After exposure to Tub at the indicated dosage for 24 h, intracellular ROS was probed with the H2DCFDA dye. The mean green fluorescence intensity represents the relative amount of ROS, which was analyzed by flow cytometry. **e** Tub significantly downregulated p-DRP1 (serine 637). **f** Mdi, a mitochondria fission inhibitor, resulted in a significant decrease in the ROS level in Tub-treated NCI-H1299 lung cancer cells. NCI-H1299 cells were treated with Tub (20 μM), Mdi (5 μM) or both for 24 h. ROS was probed with the H2DCFDA dye, and flow cytometry analysis was performed. **g** Mdi partially rescued the inhibitory effect of Tub on lung cancer cells. ***p < 0.001 vs. the indicated group.

We performed the CCK8 assay to examine the cell viability of lung cancer cells from two cell lines (NCI-H1299 and NCI-H1975) treated with Tub at a set concentration gradient. The results showed that Tub inhibited cell viability in both NCI-H1299 and NCI-H1975 cells in a dose-dependent manner (Fig. 1b), at IC50 values of 17.53 and 25.01 μM, respectively. The inhibitory effect of Tub was also confirmed by colony formation assay and CFDA SE staining assay (Fig. S2).

To determine whether the effect of Tub on cell viability was dependent on its effect on mitochondrial function, mitochondrial staining with MitoTracker was performed. The findings showed that the mitochondria in Tub-treated cells were short and thick. This implies that Tub induced the fragmentation of mitochondria in the lung cancer cells (Fig. 1c). Then, the amount of ROS was detected by H2DCFDA staining, which showed that Tub treatment resulted in significant accumulation of ROS (Fig. 1d).

Mitochondria maintain their quality by maintaining a balance between the processes of fission and fusion, and excessive fission results in an increase in the fragmentation of mitochondria and loss of its polarization^10^. Dynamin-related protein (DRP1), a small GTPase, is a critical factor that mediates mitochondrial fission. Dephosphorylation of DRP1 at serine 637 has been found to inhibit mitochondrial fission via prevention of the translocation of DRP1 to mitochondria^11^. Western blot analysis in this study showed that the level of phosphorylated DRP1 at serine 637 (p-DRP1) was significantly downregulated by Tub treatment (Fig. 1e). Additionally, the inhibition of DRP1 with its specific inhibitor Mdivi-1 (Mdi) resulted in reversal of the inhibitory effect of Tub on lung cancer cells. Thus, the results indicate that Tub-induced inhibition of the proliferation of lung cancer cells might be the result of DRP1-dependent fragmentation (Fig. 1f, g).

### Tub inhibited the late stage of autophagic flux in lung cancer cells

**Fig. 2:**
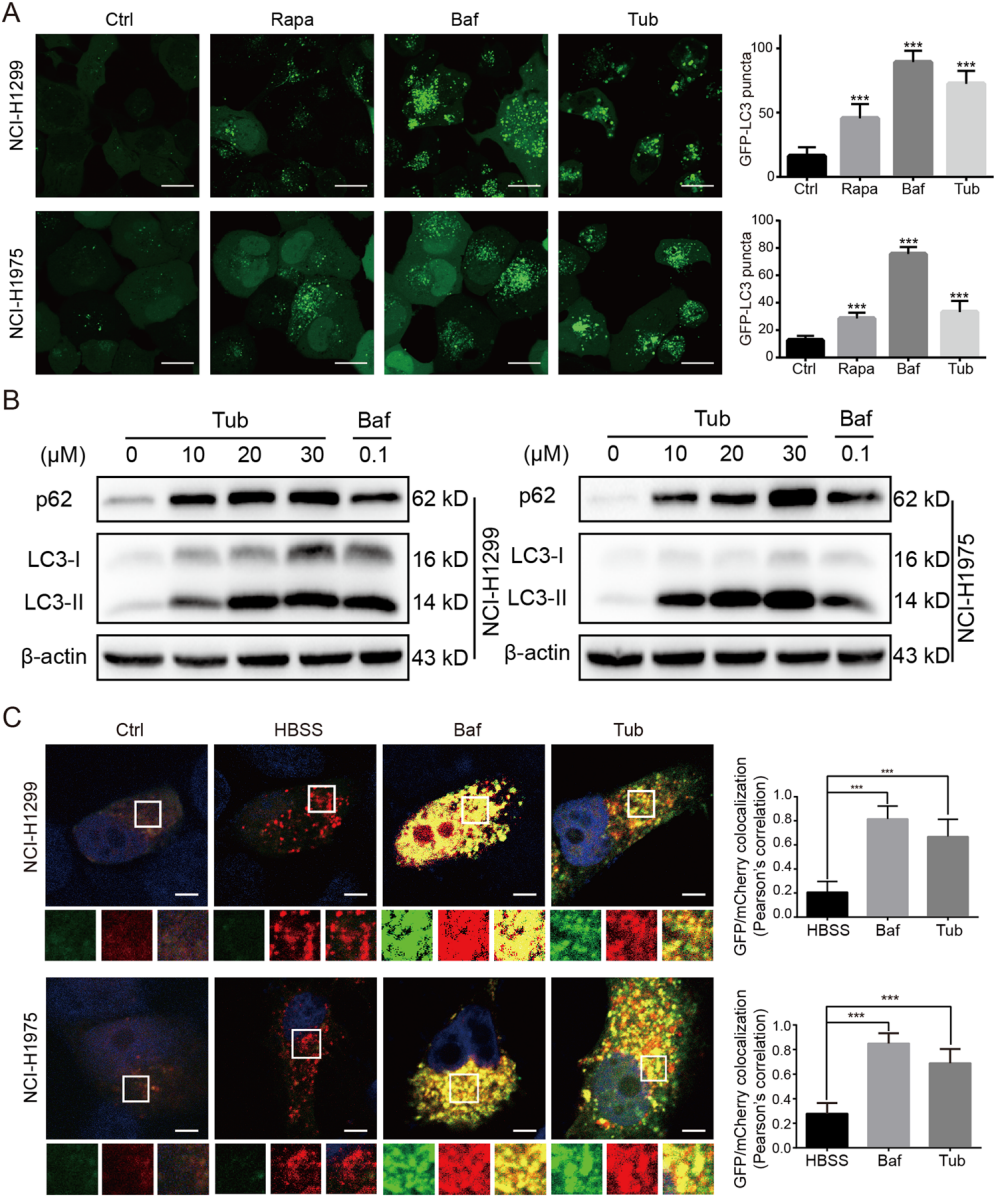
Tub induced blocking of late-stage autophagic flux in lung cancer cells. **a** Tub induced an increase in the number of GFP-LC3 puncta. GFP-LC3-overexpressing stable cell lines were treated with the vehicle, rapamycin (Rapa, 0.5 μM), bafilomycin A1 (Baf, 0.1 μM) or Tub (20 μM) for 24 h. Images of the cells were captured with a laser-scanning confocal microscope (scale bar = 20 µm). **b** Tub induced the upregulation of LC3-II and p62. **c** Lung cancer cells transfected with mCherry-GFP-LC3 tandem plasmids were treated with the vehicle, HBSS, Baf (0.1 μM) or Tub (20 μM) for 24 h. Like Baf treatment, Tub treatment also caused an increase in yellow fluorescence (creating by the merging of red and green fluorescence emitted by mCherry and GFP, respectively). The images were captured by a laser-scanning confocal microscope. The bar chart (right) represents the colocalization rate of GFP and mCherry, which was calculated with the Image J software (Scale bar = 5 µm). ***p < 0.001 vs. the indicated group.

Some of the fragmented mitochondria can achieve polarization by fusion to recover their functions, but to a large extent, the dysfunctional mitochondria are eliminated by the autophagy process^12^. In order to investigate the effect of Tub on autophagic flux in cancer cells, we established two lung cancer stable cell lines overexpressing the GFP-LC3 fusion protein, which is considered as an indicator of the number of intracellular autophagosomes. As shown in Fig. 2a, we treated the cells with Tub, vehicle (control), rapamycin (Rapa, a canonical inducer of autophagy) or bafilomycin A1 (Baf, an inhibitor of the late stage of autophagic flux). The results indicated that Tub induced a significant increase in the number of autophagosomes as compared with the vehicle group. The results were confirmed by western blot analysis, which showed the upregulation of LC3-II (a marker of autophagosomes) induced by Tub in a dose-dependent manner. The increase in the number of autophagosomes may be the result of upregulation of autophagy initiation, as observed in the Rapa group, or blocking of degradation in the late autophagic flux stage, as observed in the Baf group. To clarify the mechanism, we evaluated the expression level of p62, which is mainly degraded by autophagy. Interestingly, following Tub treatment, p62 was upregulated in a dose-dependent manner; this indicates the blockage of autophagy. Thus, another mechanism of action of Tub, apart from mitochondrial fragmentation, is the simultaneous blocking of the recycling pathway by autophagy inhibition (Fig. 2b).

To further understand the effect of Tub on autophagic flux, we transfected mCherry-GFP-LC3 plasmids into lung cancer cells and observed fluorescence emission by the fusion protein. The conformation of GFP changes in an acidic environment, and as a result, its green fluorescence emission is diminished. Therefore, under normal autophagic flux, as in the Hanks’ Balanced Salt Solution (HBSS) group, autophagosomes fuse with the low-pH lysosome and green fluorescence is poor (Fig. 2c). In contrast, in the presence of an inhibitor of lysosomal acidification, for instance, Baf, which acts as an inhibitor of V-ATPase, there is an increase in yellow fluorescence (as a result of overlap of red and green fluorescence). Consistent with this, the Tub treatment group also showed a large amount of yellow fluorescence. Thus, Tub possibly inhibited the acidification of lysosomes, as Baf did, or blocked the fusion of autophagosomes and lysosomes. Irrespective of the mechanism, the findings clearly indicate that Tub blocked late-stage autophagic flux.

### Tub impaired lysosomal acidification by inhibiting ATPase activity

**Fig. 3:**
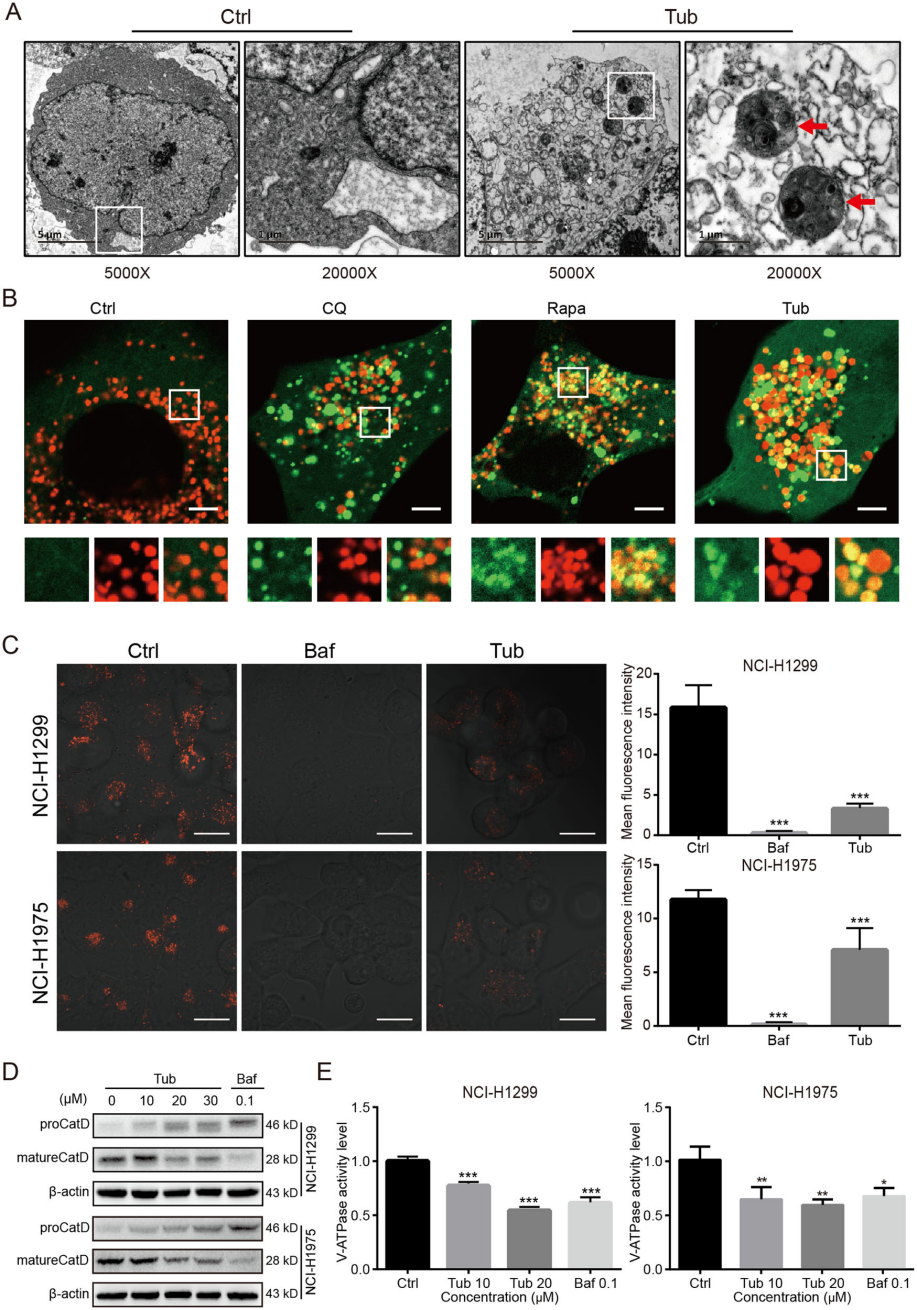
Tub induced impairment of lysosomal acidification via inhibition of V-ATPase activity. **a** The images were captured with a transmission electron microscope. The area within the white rectangle is enlarged and shown in the panels on the right. The red arrow indicates autolysosomes. **b** Colocalization of autophagosomes and lysosomes in lung cancer cells following Tub treatment. The autophagosomes are labelled by the GFP-LC3 (green fluorescence) protein and the lysosomes are labeled by LysoBrite^TM^ Red (scale bar = 5 μm). **c** Tub significantly inhibited the acidification of lysosomes in lung cancer cells. LysoTracker Red was used as a fluorescent probe for acidic lysosomes, and is an indicator of lysosomal acidity (scale bar = 20 µm). **d** Tub treatment resulted in a significant decrease in the level of mature cathepsin D. **e** Tub (at concentrations of 10 and 20 μM) significantly inhibited V-ATPase activity; the effect was similar to that of Baf (at a concentration of 0.1 μM).

To determine whether Tub inhibited late-stage autophagic flux by blocking autophagosome-lysosome fusion or suppressing lysosomal acidification, we observed subcellular organelles by transmission electron microscopy and found a high number of autolysosomes in the cells treated with Tub (Fig. 3a). The data indicate that Tub did not fully block the fusion of autophagosomes and lysosomes. Thus, the inhibitory effect of Tub on autophagy may be the result of suppression of lysosomal acidification. To confirm this, we treated cells of the stable cell line NCI-H1299^GFP-LC3^ with Tub, rapamycin, or chloroquine (an inhibitor of autophagosome-lysosome fusion), and then dyed the cells with LysoBrite for lysosomal staining. As shown in Fig. 3b, compared with chloroquine treatment, Tub resulted in the colocalization of red and green fluorescent puncta, as indicated by puncta emitting yellow fluorescence. Thus, Tub may obstruct autophagic flux by suppressing lysosomal acidification rather than by blocking autophagosome-lysosome fusion. Moreover, we also observed red fluorescence in the cells treated with Tub and stained with the LysoTracker Red DND-99 dye (Fig. 3c). Similar to Baf treatment, a canonical inhibitor of lysosomal acidification, exposure to Tub resulted in a decrease in red fluorescence; this is indicative of aberrant lysosomal acidification. An abnormal pH value is bound to impede the maturation of acid hydrolase in the lysosome. Cathepsin D is first present in the proform and is later processed and matured in the acidic lysosome. Consistent with this, our data indicated a dose-dependent reduction of mature cathepsin D with Tub treatment (Fig. 3d).

As V-ATPase is a major engine that pumps protons into lysosomes and generates an acidic environment, we hypothesized that, like Baf (which is a V-ATPase inhibitor), Tub might inhibit the activity of V-ATPase^13^. The data from the enzymatic activity assay confirmed our hypothesis, as Tub was found to significantly reduce V-ATPase activity (Fig. 3e). Taken together, the findings indicate that Tub blocks late-stage autophagic flux by inhibiting lysosomal acidification via inhibition of V-ATPase activity.

### Excessive ROS caused an increase in lysosomal membrane permeability and led to the cytosolic release of cathepsin B

As indicated by the findings so far, Tub not only induced mitochondrial fragmentation, but also simultaneously blocked the removal of dysfunctional mitochondria. This would lead to the accumulation of ROS, a byproduct of mitochondrial damage. Therefore, in this experiment, the amount of intracellular ROS was measured by H2DCFDA staining, and the results showed that Tub treatment resulted in significant ROS accumulation in lung cancer cells (Fig. 1d).

**Fig. 4:**
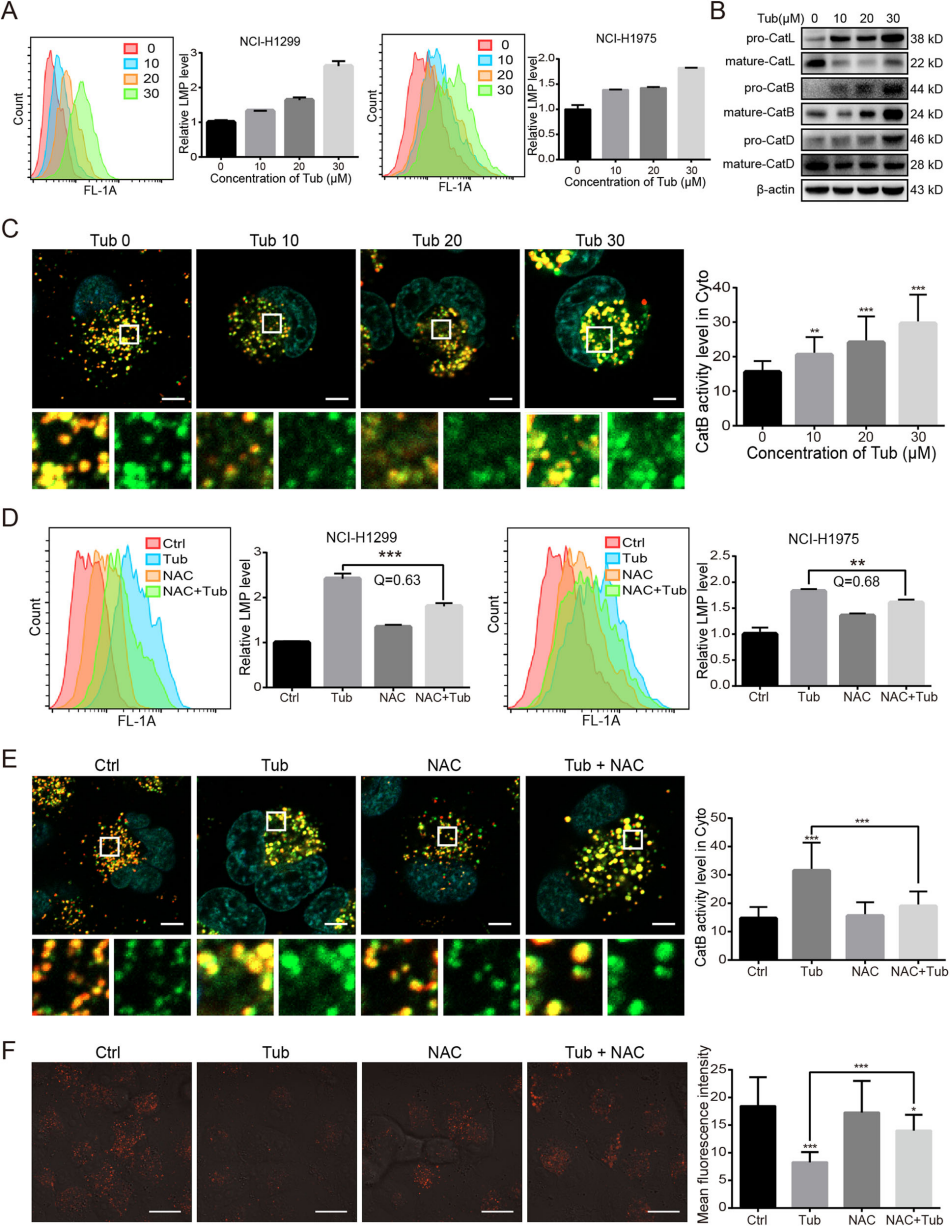
Excessive ROS results in an increase in LMP and leads to the cytosolic release of cathepsin B. **a** Tub induced an increase in LMP. After exposure to Tub for 24 h, cells were stained with AO (5 μg/mL) for 20 min. The mean fluorescence intensity in the FL1 channel represents the extent of LMP. The bar chart was drawn from the flow cytometry results. **b** The level of cathepsins in the cytoplasm of lung cancer cells after Tub treatment. Cytosolic protein was extracted using a commercially available kit and subjected to western blot analysis. **c** The activity of cytosolic cathepsin B was significantly upregulated following Tub treatment. Cathepsin B activity is labelled by green fluorescence; lysosomes are labeled by red fluorescence (LysoBrite^TM^ Red); and nuclei are labelled by blue staining with Hoechst 33342. The green fluorescence intensity in the cytosolic area represents the activity of cytosolic cathepsin B. Cathepsin B activity was quantified in more than 30 cells for each group with the help of the Image J software, and the data are presented in the right bar chart (scale bar = 5 µm). **d** Elimination of ROS by NAC (an ROS scavenger) reversed the increase in LMP induced by Tub. Lung cancer cells were treated with Tub (20 μM), NAC (ROS scavenger, 2 mM) or both Tub and NAC for 24 h. LMP was determined by AO staining for 20 min. A Q value of <0.85 indicates that NAC and Tub have an antagonistic effect on each other. **e** NAC treatment led to decreased activity of cytosolic cathepsin B in Tub-treated lung cancer cells (for details, see the description for panels **c** and **d**) (scale bar = 5 µm). **f** NAC partially reversed the lysosomal acidification in Tub-treated lung cancer cells. LysoTracker Red was used as a fluorescent probe for acid lysosomes (scale bar = 20 µm). *p < 0.05, **p < 0.01, ***p < 0.001 vs. the indicated groups.

The accumulation of intracellular ROS can increase lysosomal membrane permeability (LMP); therefore, we stained lung cancer cells with acridine orange (AO) to examine LMP. AO molecules are protonated in a low-pH environment, and therefore, AO shows a preference for accumulation in lysosomes, where it emits red fluorescence^14^. When LMP increases, AO leaks from the lysosomes into the cytoplasm, wherein it can be excited to emit green fluorescence. By measuring the intensity of green fluorescence by flow cytometry, the relative level of LMP can be determined. As seen in Fig. 4a, the level of LMP increased after Tub treatment in a dose-dependent manner.

The increase in LMP might also be the result of the release of cathepsins from lysosomes into the cytoplasm^15^; therefore, we extracted the cytosolic fraction of the cells to examine the amount of major cathepsins by western blot analysis. As shown in Fig. 4b, the level of cathepsin B significantly increased in the cytosolic fraction of lung cancer cells after Tub treatment. In addition, the activity of cytosolic cathepsin B was also evaluated through an enzymatic reaction; active cathepsin B can cleave the Ac-RR-AFC substrate and generate a green fluorescent product (Fig. 4c). The LysoBrite dye (red) was also added for labeling lysosomes. With the help of the Image J software, the intensity of green fluorescence in the cytosolic area was determined. Tub induced a significant increase in green fluorescence intensity; this indicates an increase in cathepsin B activity in the cytosolic fraction of lung cancer cells.

To determine whether excessive ROS is essential for LMP increase and subsequent cathepsin B release, we treated the cancer cells with acetylcysteine (NAC), a well-known ROS scavenger. The NAC treatment reversed the LMP increase as well as increased cytosolic cathepsin B activity in Tub-induced lung cancer cells (Fig. 4d, e). Theoretically, the increase in LMP might also lead to the leak of lysosomal protons, which might form a feedback loop and result in the aggregation of aberrant lysosomal acidification^16,17^. Consistent with our hypothesis, NAC significantly reversed the abnormal lysosomal acidification caused by Tub in lung cancer cells (Fig. 4f).

Altogether, the findings indicate that Tub-induced accumulation of ROS was critical for the increase in LMP and subsequent release of lysosomal content, including cathepsin B and (possibly) protons.

### Cytosolic cathepsin B promoted mitochondrial outer membrane permeability

**Fig. 5:**
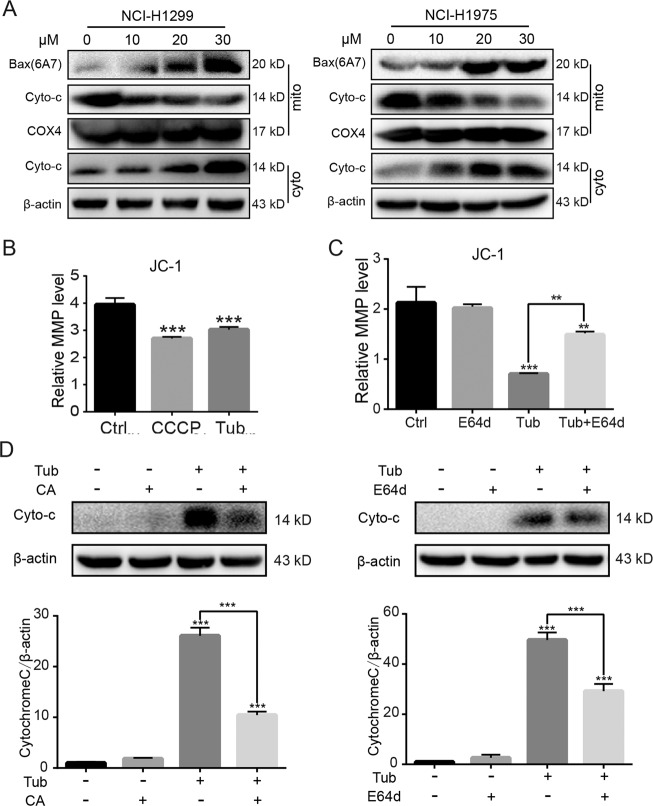
Cytosolic cathepsin B induced an increase in MOMP. **a** Tub induced upregulation of conformation-changed Bax and downregulation of cytochrome C in mitochondria, and an increase in cytochrome C in the cytoplasm. The mitochondrial and cytosolic fractions were extracted and subjected to western blot analysis. **b** Tub induced a significant increase in MOMP. NCI-H1975 cells were treated with Tub (20 μM) or CCCP (10 μM) for 24 h; CCCP was used as a positive control, as CCCP can induce the depolarization of mitochondria. Mitochondrial membrane potential (MMP) was measured with the JC-1 kit. **c** E64d, a cathepsin B inhibitor, significantly reversed the increase in MOMP induced by Tub. NCI-H1975 cells were treated with Tub (20 μM), E64d or both Tub and E64d for 24 h. The mitochondrial membrane potential (MMP) was measured with the JC-1 kit. **d** Treatment with E64d (20 μM) or CA (CA-074 methyl ester, 10 μM) (both inhibitors of cathepsin B) for 24 h resulted in a significant reduction in cytosolic cytochrome C in Tub-treated NCI-H1299 cells. **p < 0.01, ***p < 0.001 vs. the indicated groups.

It is known that the proapoptotic proteins Bax (which is important for pore formation in the mitochondrial outer membrane) and cytochrome C (which is translocated to the cytoplasm) play a role in triggering caspase-dependent apoptosis^18^. Accordingly, in this study, significant upregulation of Bax in the mitochondrial fraction and cytochrome C in the cytosolic fraction was observed following Tub treatment of cancer cells (Fig. 5a). We measured the mitochondrial membrane potential, a hallmark of mitochondrial outer membrane permeability (MOMP), with the JC-1 assay. Carbonyl cyanide m-chlorophenyl hydrazone (CCCP) treatment was used as a positive control, as CCCP can induce depolarization of mitochondria. The results showed that exposure to Tub resulted in a decrease in mitochondrial membrane potential, as observed with CCCP treatment (Fig. 5b). On the other hand, addition of the cathepsin B inhibitor aloxistatin (E64d) reversed the Tub-induced decrease in mitochondrial membrane potential (Fig. 5c). Moreover, addition of a cathepsin B inhibitor (CA-047 methyl ester or E64d) also decreased the level of cytosolic cytochrome C (Fig. 5d). These results indicate that the Tub-induced decrease in MOMP was dependent on the leakage of cathepsin B from the lysosome.

### Inhibition of cathepsin B and ROS clearance reversed Tub-induced apoptosis in lung cancer cells

**Fig. 6:**
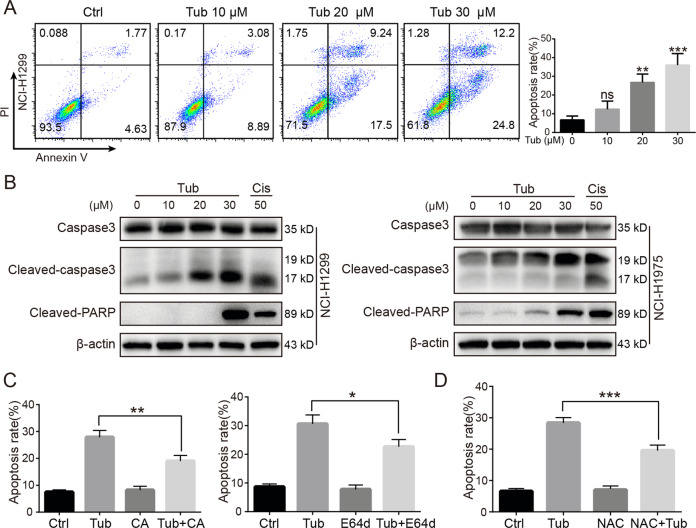
Cathepsin B inhibition and ROS clearance reversed Tub-induced apoptosis in lung cancer cells. **a** The apoptosis rate of NCI-H1299 cells after treatment with Tub at the indicated concentrations, as demonstrated by Annexin-V/PI staining and flow cytometry analysis. **b** The cleaved forms of PARP and Caspase 3 were upregulated in lung cancer cells following Tub treatment. **c** The cathepsin B inhibitor, in part, reversed the apoptosis in NCI-H1299 cells treated with Tub (the treatment details can be found in Fig. 5). **d** NAC (a ROS scavenger), in part, rescued the Tub-induced apoptosis in NCI-H1299 cells. *p < 0.05, **p < 0.01, ***p < 0.001 vs. the indicated groups.

Tub-induced cancer cell apoptosis was examined with the Annexin-V/PI assay. As shown in Fig. 6a, Tub induced lung cancer cell apoptosis in a dose-dependent manner. The cellular phenotype was confirmed by upregulation of apoptotic markers, including cleaved-PARP and cleaved-caspase 3 (Fig. 6b). Neutralization of ROS using NAC (an ROS scavenger) or inhibition of cathepsin B with its specific inhibitors E64d or CA reversed Tub-induced apoptosis in a significant way (Fig. 6c, d). Thus, ROS and cathepsin B seem to be critical upstream players in the induction of apoptosis by Tub.

### Tub inhibited the in vivo growth of lung cancer cells

**Fig. 7:**
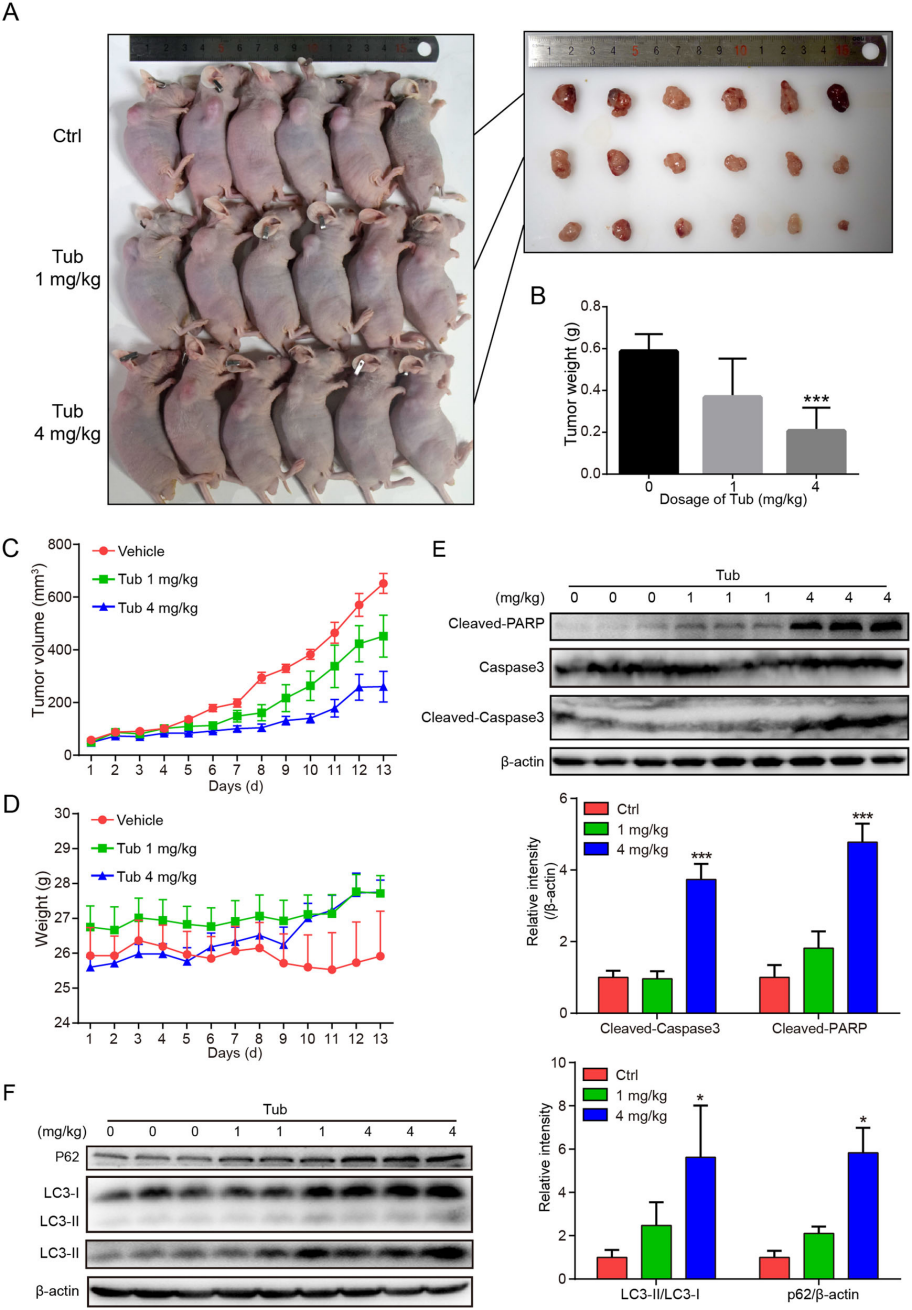
Tub induced inhibition of xenografted lung tumor growth. **a** Subcutaneous tumors were induced in nude mice by subcutaneous injection of NCI-H1299 cells in the right flank. When the tumors reached a diameter of 0.1 mm^3^, the mice were divided into three groups (*n* = 6 for each group) and subjected to intraperitoneal Tub administration at the indicated doses. **b** The tumors were resected and weighed on the 13th day of drug treatment; the 4 mg/kg dose was associated with a significant decrease in tumor weight as compared to the control group. **c** Tumor volume was measured every day, and was lower in the Tub-treated groups than in the control group. The displayed data were recorded from Day 1 after Tub treatment and presented as mean ± standard error. **d** The body weight of the mice was measured every day, and Tub treatment did not result in a decrease in the body weight. Data are presented as mean ± standard error. **e, f** The xenografted tumors were ground for protein extraction and western blot analysis of autophagy and apoptosis markers, which were significantly upregulated in the 4 mg/kg Tub group as compared to the control group. *p < 0.05, ***p < 0.001 vs. the indicated groups.

The anticancer effect of Tub was verified under in vivo conditions. NCI-H1299 cells were subcutaneously injected into the right flank of nude mice, which were randomly divided into three groups treated with vehicle, 1 mg/kg Tub or 4 mg/kg Tub by intraperitoneal injection, respectively. On the 13th day of drug treatment, the mice were sacrificed and the tumors were weighed. The tumor size was apparently smaller than that in the control group (Fig. 7a), and the tumor weight in the 4 mg/kg Tub group was significantly lower than that in the control group (Fig. 7b). These inhibitory effects of Tub were dose dependent. In addition, we measured the tumor volume every day, and the growth curve of the tumors indicated a lower growth rate in the Tub group than in the control group. Besides, tumor volume in the Tub groups was significantly lower (*p* < 0.05) than that in the vehicle group since Day 8 (Fig. 7c). Additionally, the body weight of the mice was measured every day, and Tub treatment did not result in a decrease in the body weight of the mice (Fig. 7d). Further, the expression level of cleaved-PARP, cleaved-caspase 3, and the autophagy markers LC3-II and p62 was significantly upregulated in tumors following Tub treatment (Fig. 7e, f). Consistent with the in vitro results, the in vivo results indicate that Tub-induced apoptosis may be brought about by blocking of autophagic flux.

## Discussion

**Fig. 8:**
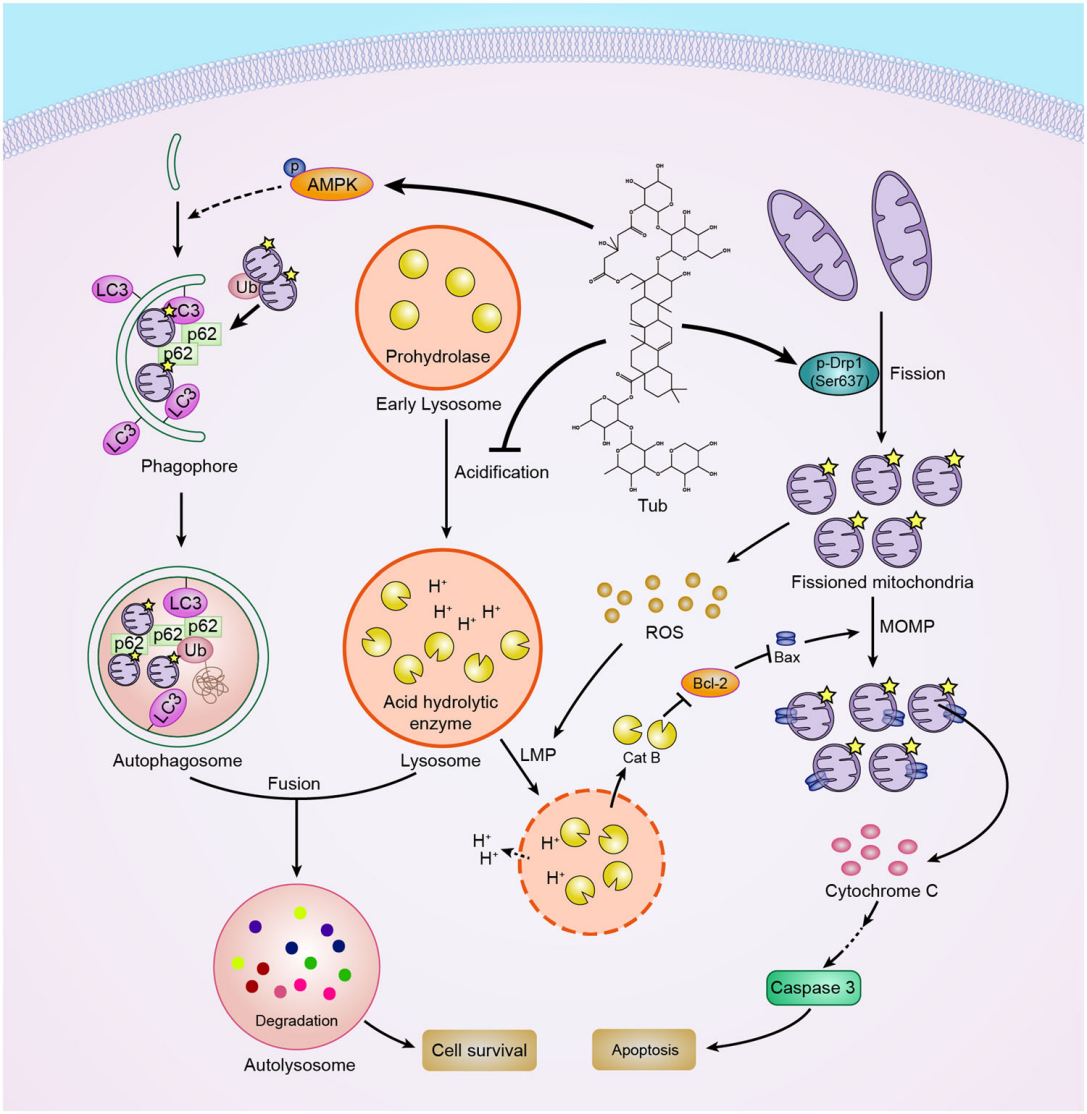
Schematic representation of the proposed mechanisms underlying the antitumor effect of Tub in lung cancer cells. As shown in the figure, Tub inhibits the late stage of autophagy flux by inhibiting the acidification of lysosomes (left). Additionally, Tub promotes mitochondrial fission and fragmentation, thereby leading to ROS accumulation (right). The accumulated ROS cannot be removed due to the blockage of autophagic flux; this causes further damage to the lysosomal membrane and leads to cathepsin B leakage from lysosomes. Cathepsin B present in the cytoplasm subsequently causes an increase in MOMP. This is accompanied by cytosolic cytochrome C-triggered caspase-dependent apoptosis. Thus, the antitumor effect of Tub is characterized by a positive feedback loop that prominently involves ROS.

In this study, we have demonstrated that the traditional Chinese antitumor agent Tub efficiently killed lung cancer cells through the induction of apoptosis under both in vitro and in vivo conditions. Additionally, through in-depth in vitro experiments, we investigated in great detail the potential mechanisms underlying the anticancer effects of Tub and have provided a schematic representation of the mechanisms and interactions in Fig. 8. We found that one of the mechanisms is the fragmentation of mitochondria through the promotion of DRP1-mediated mitochondrial fission. Another important mechanism is the blocking of late-stage autophagic flux via impairment of lysosomal function through the inhibition of V-ATPase. This mechanism blocks the removal of dysfunctional mitochondria and results in the accumulation ROS. Excessive ROS accumulation causes damage to lysosomal membranes and increases LMP, which in turn leads to the leakage of cathepsin B. It has been reported that cathepsin B can promote the degradation of Bcl-2 by cleaving it^19^. This leads to translocation of Bax to mitochondria and upregulation of MOMP, and this is followed by the release of cytochrome C. This finally led to irreversible apoptosis. Thus, the cancer cell killing effect of Tub is enhanced through the formation of a positive feedback loop.

Mitochondria in cancer cells undergo regular cycles of fission and fusion; thus, their optimal functioning is maintained through autophagic pathways. The Tub-induced upregulation of fission disrupted this balance and led to an increase in the fragmentation of mitochondria. This resulted in the accumulation of excessive ROS, such as superoxide anions, hydrogen peroxide, and hydroxyl free radicals^19,20^. Cancer cells usually exhibit potent adaptive capacity through upregulation of autophagy pathways. In this study, the level of phosphorylated adenosine 5ʹ-monophosphate-activated protein kinase (AMPK) was upregulated in lung cancer cells following Tub treatment (Fig. S1). AMPK is a canonical factor that promotes autophagy; therefore, the rescue pathway was probably initiated in the cancer cells on exposure to Tub. Thus, the increase in autophagosomes observed in the lung cancer cells treated with Tub might be the result of both initiation of autophagy and blocking of late-stage autophagic flux. Similar results have been reported by Feng et al. in cervical cancer cells^21^. Their findings indicated that Tub blocked autophagic flux by impairing the maturation of cathepsin D, but they did not examine in detail the apoptotic mechanisms triggered by Tub or the effect of Tub on lysosomal enzymes^21^. In this study, we have made important contributions in this regard by providing in-depth data to show that Tub was able to impair lysosomal acidification in lung cancer cells. Further, our data also indicated that exposure to Tub caused a decrease in V-ATPase activity, which might explain the aberrant pH in lysosomes in the Tub-treated cells. Moreover, we have shown that LMP induced by ROS can cause lysosomal contents, including protons, to leak out and thus contribute to augmenting the pH in lysosomes. Since the mitochondria, the main machinery that produce ATP, was damaged, and ATP is the substrate of V-ATPase, we hypothesized that the level of intracellular ATP might decrease and possibly contribute to the impotence of the proton pump. However, our data did not show a decrease in the ATP level in lung cancer cells following Tub treatment (Fig. S2). This implies that Tub did not impair lysosomal acidification by interfering with the generation of ATP.

Our present findings indicate that ROS plays a pivotal role in the induction of apoptosis in Tub-treated lung cancer cells through induction of MOMP. Excessive ROS may activate the mitogen-activated protein kinase (MAPK) pathway, which is involved in the regulation of MOMP^22^. It has been found that phosphorylation of Bax mediated by JNK and p38 kinase can lead to its activation, and this can consequently lead to Bax translocation and MOMP^23^. To determine whether ROS directly induced MOMP, we performed a series of experiments. Our data showed that Tub induced the upregulation of p-JNK and p-p38 (Fig. S4A). However, elimination of ROS by NAC treatment did not reverse the level of phosphorylation of JNK and p38 (Fig. S4B). Thus, ROS may not be an essential factor for activation of the MAPK pathway. In addition, inhibition of JNK and p38 by specific inhibitors did not reverse the killing effect of Tub (Fig. S4C). These data indicate that ROS accumulation induced by Tub may not induce MOMP via a direct pathway. Eventually, we demonstrated that Tub induced MOMP and, subsequently, apoptosis in lung cancer cells via a lysosomal-dependent pathway wherein ROS played a critical role.

Apoptosis is the self-killing pathway in cells, and in comparison, autophagy is the self-eating and recycling process. The crosstalk between them is attractive but complex^24,25^. Our in-depth research findings may be helpful to better understand the relationship between autophagy and apoptosis, especially in cancer cells. More importantly, our promising findings with regard to the potent anticancer effect of Tub makes an important contribution to research on lung cancer chemotherapy.

## Materials and methods

### Cell culture and materials

The human lung cancer cell lines NCI-H1299 (ATCC® CRL-5803™) and NCI-H1975 (ATCC® CRL-5908™) were obtained from American Type Culture Collection (ATCC, Rockville, MD, USA). All cells were cultured in Dulbecco’s modified Eagle’s medium (DMEM) supplemented with 10% fetal bovine serum and 100 U/mL penicillin/streptomycin at 37 °C in a 5% CO_2_ atmosphere. All reagents for the cell culture were purchased from Gibco Life Technologies (Grand Island, NY, USA), unless otherwise stated.

Tubeimoside I (Tub, T2715) was purchased from Targetmol (Boston, MA, USA). Bafilomycin A1 (Baf, S1413), acetylcysteine (NAC, S1623) and rapamycin (Rapa, S1039) were purchased from Selleckchem (Houston, TX, USA). Primary antibodies against β-actin (3700), LC3B (3868), p62 (88588), cathepsin B (31718), cathepsin D (2284), Caspase 3 (9662), p-DRP1 (ser616) (3455), p-DRP1 (ser637, (4867), cytochrome C (11940), COX 4 (4850), and cleaved-PARP (5625) were obtained from Cell Signaling Technology (Boston, MA, USA). Cathepsin L (AF952) was obtained from R&D Systems Inc. (Minneapolis, MN, USA). Anti-Bax (6A7) antibody was obtained from Santa Cruz Biotechnology (Dallas, TX, United States). The secondary antibodies peroxidase-labeled anti-mouse IgG (AS004) and anti-rabbit IgG (AS014) were obtained from Abclonal (Wuhan, China).

### Cell viability assay

Cells were seeded in 96-well plates and incubated overnight before treatment with the indicated compounds for 24 h. Cell proliferation was measured using Cell Counting Kit-8 (CCK8, CK04; Dojindo Laboratories, Kumamoto, Japan) following the manufacturer’s instruction. Briefly, the medium was discarded from each well, and 100 µL of CCK8 solution (10-fold dilution in medium) was added. Then, the plates were incubated for 2 h at 37 °C, and the absorbance of each well was measured at 450 nm.

### Western blot analysis

Cells were lysed using lysis buffer containing protease inhibitors. The protein samples were separated by 12% sodium dodecyl sulfate-polyacrylamide gel electrophoresis (SDS-PAGE) and transferred onto PVDF membranes (0.22 µm; Roche, Branchburg, NJ, USA). Then, the membranes were blocked for 2 h with 5% nonfat dry milk and incubated overnight with the indicated primary antibody (1:1500 dilution) at 4 °C. Subsequently, the membrane was washed thrice with TBST (0.05% Tween 20 in Tris-buffered saline) and incubated with the secondary antibodies (1:4000 dilution) at room temperature. Immunoreactive bands were obtained with enhanced chemiluminescence (ECL, WBLS0500; Millipore, Burlington, MA, US). The intensity of the bands was quantified by the Gel-Pro Analyzer program (Media Cybernetics, Silver Spring, MD, USA).

### Autophagosome and lysosome colocalization

NCI-H1299 cells stably expressing LC3-GFP were seeded in 15-mm dishes with a glass bottom and cultured for 24 h. The cells were treated with the indicated compounds for 24 h. Then, the cells were treated with LysoBrite^TM^ Red (22645, AAT Bioquest, CA, USA) for 20 min at 37 °C in DMEM for lysosome staining. Following this, the medium was removed and the cells were washed three times with PBS. Images were captured with a laser confocal scanning microscope equipped with a ×63 objective lens (LSM 800; Carl Zeiss, Jena, Germany).

### V-ATPase activity detection assay

A V-ATPase assay kit (GMS50244) was purchased from Genmed Scientifics (Arlington, MA, USA). The effects of Tub or Baf on V-ATPase activity were determined according to the manufacturer’s instructions. Cells were seeded in a 6-well plate and incubated for 24 h before drug treatment. Then, protein extraction was performed, and the protein concentration was measured and adjusted to 2 μg/μL. The protein fraction obtained from each group was aliquoted into two separated tubes (for total and nonspecific ATPase activity detection) and mixed with the reagents provided by the kit (Reagents A-H). The absorbance of the mixtures from each tube was measured at 340 nm immediately, and measurements were taken again after a 10-min interval. All the measurements were taken at 37 °C. V-ATPase activity was calculated by subtracting nonspecific ATPase activity from total ATPase activity. The activity of each group was normalized with the DMSO (Ctrl) group.

### MitoTracker and LysoTracker staining

Cells were seeded in 15-mm confocal dishes and cultured for 24 h. Then, the cells were treated with different concentrations of Tub for 24 h. The medium was discarded, and the cells from each group were washed with PBS three times. The cells were then stained with MitoTracker Red CMXRos (200 nM; Cell Signaling Technology, Boston, MA, USA) or LysoTracker Red DND-99 (50 nM, L7528; Invitrogen, Carlsbad, CA, USA) for 20 min at 37 °C in the dark and washed three times with PBS. Images were captured with a laser-scanning confocal microscope equipped with an argon laser (excitation wavelength: 555 nm) and a ×63 objective lens (LSM 800; Carl Zeiss, Jena, Germany).

### Intracellular ROS detection

NCI-H1299 or NCI-H1975 cells were plated in 6-well plates, cultured overnight, and treated with different concentrations of Tub for 24 h. The cells were collected, washed with PBS, and stained with 10 µM H2DCFDA (D399; Thermo Fisher Scientific, Waltham, MA, USA) for 20 min at 37 °C in the dark. Each sample was washed and resuspended in 500 μL HBSS. For each sample, 10,000 cells were analyzed using the FL1 channel of a BD Accuri C6 flow cytometer (BD Pharmingen, San Diego, CA, USA).

### Autolysosome detection

NCI-H1299 cells were plated on 10-cm dishes at a density of 2 × 10^6^ cells/dish. The cells were treated with vehicle or Tub for 24 h. After treatment, the cells were collected and fixed in 2.5% glutaraldehyde overnight, and then incubated with osmium tetraoxide for 2 h at 4 °C. Specimens were embedded in epoxy resin. Sections of 100-nm thickness were prepared and stained with uranyl acetate and lead citrate for observation of autolysosomes. Sections were imaged using a transmission electron microscope (Hitachi HT7700; Hitachi, Tokyo, Japan).

### Lysosomal membrane permeability detection

NCI-H1299 or NCI-H1975 cells were plated on 6-well plates overnight and treated with different concentrations of Tub for 24 h. The cells were collected, washed with PBS, and stained with acridine orange (5 µg/mL AO, A6014; Sigma Biotechnology, St. Louis, MO, USA) at 37 °C for 20 min. Each sample was washed and resuspended in 500 μL HBSS. For each sample, 10,000 cells were analyzed using the FL1 channel of a BD Accuri C6 flow cytometer.

### Cathepsin B activity detection

Cells were seeded in 15-mm small confocal dishes and cultured for 24 h. Then, the cells were treated with different concentrations of Tub for 24 h. After washing three times with PBS, each sample was incubated with Ac-RR-AFC (K140; BioVision Inc., CA, USA) at 37 °C for 1 h. Then, the cells were stained with 5 µg/mL Hoechst 33342 and LysoBrite^TM^ Red (1:2000 dilution, 22645; AAT Bioquest, CA, USA) at 37 °C for 20 min. Images were captured with a confocal laser-scanning microscope equipped with an argon laser (excitation wavelength: 405 nm, emission wavelength: 505 nm) and a ×63 objective lens. Then, the green fluorescence intensity in the cytosolic area was calculated using the threshold plugin in the Image J software (US National Institutes of Health, Bethesda, MD, USA).

### Mitochondrial membrane potential measurement

Cells seeded in 6-well plates, cultured overnight, and treated with different concentrations of Tub for 24 h. The cells were harvested and resuspended in 500 µL DMEM. Each group of cells was stained with the JC-1 dye according to the instruction manual (551302; BD Pharmingen, San Diego, CA, USA). Then, the plate was incubated at 37 °C in the dark for 20 min. The cells were then washed with precooled 1× JC-1 buffer twice. The treated cells were resuspended in 500 µL 1× JC-1 buffer and analyzed using the BD Accuri C6 flow cytometer.

### Annexin-V/PI apoptosis detection assay

Cells were seeded into six-well plates. After overnight culturing, the cells were treated with different compounds (as indicated for the different experiments) for 24 h. The cells were collected, washed with PBS, and resuspended in 600 µL Annexin-V-binding buffer. Then, Annexin-V-FITC (3 µL) was added to each sample, and the plate was incubated at 37 °C in the dark for 20 min. Following this, 5 µL PI was added to the samples for an additional 5 min of incubation. For each sample, 10,000 cells were analyzed using the FL1 and FL3 channels of the BD Accuri C6 flow cytometer.

### Cytosolic fraction and mitochondrial fraction extraction

The cytosolic fraction and mitochondrial fraction of the treated cells were extracted with a cytosolic protein extraction kit (BB-3113) and a mitochondrial protein extraction kit (BB-3171), which were purchased from BestBio (Shanghai, China). Treated cells were collected and washed twice using PBS. Following this, 500 µL of mitochondrial or cytoplasmic protein extraction reagent was added to each sample, which was incubated on ice for 20 min. The samples were vortexed every 5 min. Cytoplasmic protein was extracted by centrifugation at 25,000 × *g* for 15 min, while mitochondrial protein was extracted by centrifugation at 11,000 × g for 20 min. The mitochondrial proteins were lysed in 1× loading buffer. The cytoplasmic proteins were quantified using a BCA protein assay kit (MA0082; Meilunbio, Dalian, China).

### Human lung cancer xenografting

The experiments using nude mice was approved by the Animal Ethics Committee at Guangzhou University of Chinese Medicine (Approval No. 20181230001). Five-week-old male BALB/c nude mice with body weights ranging from 18 to 22 g were purchased from the Laboratory Animal Center of Guangzhou University of Chinese Medicine and housed in a standard animal laboratory. They were given ad libitum access to sterilized water and food, and after a 1-week acclimation period, NCI-H1299 cells (5 × 10^6^ in 200 µL) were subcutaneously injected into the right flanks of the mice. The mice were examined every day. When the tumors reached a diameter of 0.1 mm^3^, the mice were randomly divided into three groups (*n* = 6 each) that were treated with vehicle (saline solution), 1 mg/kg Tub, or 4 mg/kg Tub every day. The investigator was blinded to the group allocation during the experiment. Tumor volume was measured every day after grouping. Then, the mice were anesthetized and sacrificed on the 13th day of drug treatment, and the tumors were dissected and weighed.

### Statistical analysis

All experiments were performed in triplicate (at the least). The investigator was blinded to the group allocation during when assessing the outcome. The sample size was chosen to ensure adequate power to detect a pre-specified effect size according to the previous reports^26,27^. Data are shown as mean ± S.D. Multiple comparisons were conducted using one-way ANOVA. If the variance is similar between the groups, LSD (Least-significant difference) method is chosen for comparison, otherwise the Games–Howell method is required. The level of significance was set at *p* < 0.001, *p* < 0.01, and *p* < 0.05. For drug combination studies, the Q value was derived using the following equation: E_ab_/(E_a_ + E_b_ − E_a_ × E_b_), where E_a_ and E_b_ represent the effects of drugs a and b, respectively, and E_ab_ represents the combined effect of the two drugs. A Q value > 1.15 indicates a synergistic relationship; a Q value between 0.85 and 1.15, an additive relationship; and a Q value < 0.85, an antagonistic relationship^28^.

## Conclusions

In summary, Tub exerted anticancer effects in lung cancer cells via inhibition of late-stage autophagy and induction of apoptosis through lysosomal-dependent pathways. The current study focused on the mechanisms of autophagy inhibition and apoptosis induced by Tub and the crosstalk between them. The findings indicated that Tub could induce an effective apoptotic effect in tumor cells by promoting lysosomal- and mitochondrial-dependent apoptosis and simultaneously inhibiting the late stage of autophagic flux. Thus, based on the present findings, Tub has immense potential as a new therapeutic agent for the treatment of lung cancer.

## Supplementary information

Supplementary materials and methods

Figure S1. Tub activated the AMPK pathway.

Figure S2. Tub inhibited lung cancer cell proliferation.

Figure S3. Tub did not decrease the ATP level in NCI-H1299 cells.

Figure S4. Tub activated the MAPK pathway, but this pathway was not associated with Tub-induced lung cancer cell inhibition.
