# Supplementary material for: Tubeimoside I-induced lung cancer cell death and the underlying crosstalk between lysosomes and mitochondria
Source: Cell Death Dis. 2020 Aug 26;11(8):708. doi: 10.1038/s41419-020-02915-x (PMC7449972; doi:10.1038/s41419-020-02915-x)
Supplement: Supplementary file 1 — Supplementary materials and methods [file 41419_2020_2915_MOESM1_ESM.doc]

**Supplementary materials and methods**

**1. Materials**

Tubeimoside I (Tub, T2715) was purchased from Targetmol (Boston, MA, USA). AS601245 (HY-11010) was purchased from MedchemExpress (Monmouth Junction, NJ, USA). Acetylcysteine (NAC, S1623), SP600125 (S1460), and SB203580 (S1076) were purchased from Selleckchem (Houston, TX, USA). Primary antibodies against β-actin (3700), AMPK (2603), p-AMPK (2535), p38 (8690), p-p38 (4511), JNK (9258), and p-JNK (9622) were obtained from Cell Signaling Technology (Boston, MA, USA). The CFDA-SE cell proliferation detection kit (C0051) and ATP detection kit (S0027) were purchased from Beyotime Institute of Biotechnology (Shanghai, China).

**2. Colony formation assay**

Cells were plated in 6-well plates and treated with the indicated compounds for 24 h. Then, the medium was removed, and colonies were washed with PBS and fixed with 4% paraformaldehyde for 20 min. The colonies were stained with crystal violet, and were photographed manually.

**3. CFDA-SE cell tracer assay**

Cells were labeled with CFDA-SE, and planted in 6-well plates and incubated overnight. Then, the cells were treated with different compounds for 24 h. The cells were harvested and washed with PBS. Finally, the cells were resuspended in HBSS for detection. Fluorescence intensity was measured on a BD Accuri^TM^ C6 flow cytometer (BD Pharmingen, San Diego, CA, USA).

**4. Intracellular ATP level detection**

The ATP level in NCI-H1299 cells was detected with an ATP detection kit according to the manufacturer’s instructions. The medium in the 6-well plate was discarded, and the colonies formed were rinsed with PBS. Then, a lysis buffer was added into each well at a volume of 200 μL. The samples were lysed on ice and collected after 15 min. After this, the samples were centrifuged at 12000 g for 5 min at a temperature of 4°C, and the supernatant was used for subsequent experiments. ATP standard solution was diluted to the following concentration gradient (0.01, 0.03, 0.1, 0.3, 1, 3, and 10 μM). The working solution for ATP detection was added to a 96-well plate (100 μL/well). Then, 20 μL of the samples or the standard solution was added to each well. The solution was mixed quickly and the RLU value was measured with a chemiluminometer. The concentration of ATP was calculated from the standard curve, and the protein concentration of the samples was determined using a BCA protein assay kit. The concentration of ATP in each sample was normalized by dividing by the concentration of the protein .

**Supplementary Figure Legends**

**Figure S1. Tub activated the AMPK pathway.** NSCLC (lung cancer) cells were treated with Tub at the indicated concentrations, and the indicated proteins were detected by western blot assay.

**Figure S2. Tub inhibited lung cancer cell proliferation. (A)** NSCLC (lung cancer) cells were treated with Tub at the indicated concentrations, and the colony formation assay was performed to determine the effect of Tub on NSCLC cell proliferation. Briefly, cells were seeded in 6-well plates, cultured for 2 days, and treated with the indicated compounds for 3 days. Then, the medium was removed, and the colonies formed were rinsed with PBS and fixed with 4% paraformaldehyde (PFA) for 20 min. The colonies were then stained with crystal violet and photographed manually. **(B)** NSCLC cells were stained with CFDA SE and seeded in a six-well plate at a density of 3 × 10^6^ cells/well. Then, the cells were treated with Tub at the indicated concentrations for 24 h. The higher the cell proliferation, the weaker is the mean fluorescence intensity. The mean fluorescence intensity was detected by flow cytometry at the FL-1 channel.

**Figure S3. Tub did not decrease the ATP level in NCI-H1299 cells.** NCI-H1299 cells were treated with Tub at the indicated concentrations. The ATP level in NCI-H1299 cells was detected with an ATP detection kit according to the manufacturer’s instruction.

**Figure S4. Tub activated the MAPK pathway, but this pathway was not associated with Tub-induced lung cancer cell inhibition.** **(A)** NSCLC (lung cancer) cells were treated with Tub at the indicated concentrations, and the indicated proteins were detected by western blot assay. **(B)** ROS clearance by NAC did not attenuate the activation of the MAPK pathway. **(C)** AS601245 and SP600125 were JNK inhibitors, and SB203580 was a p38 inhibitor. CCK8 assay showed that JNK and p38 inhibition did not reverse the Tub-induced inhibition of cell viability.
