# Supplementary figures and images for: Tubeimoside I-induced lung cancer cell death and the underlying crosstalk between lysosomes and mitochondria
Source: Cell Death Dis. 2020 Aug 26;11(8):708. doi: 10.1038/s41419-020-02915-x (PMC7449972; doi:10.1038/s41419-020-02915-x)

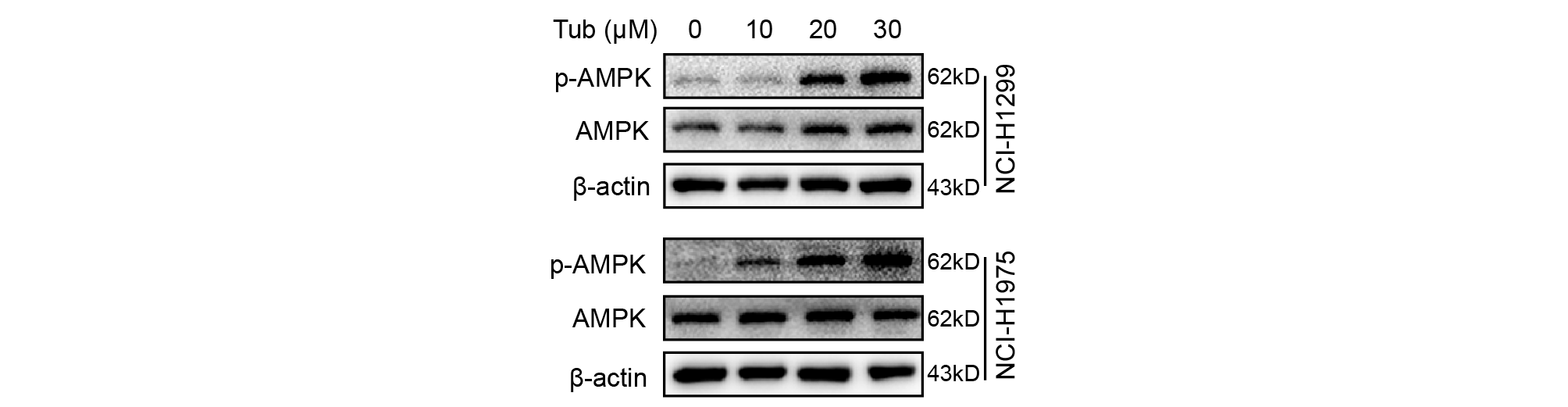

Supplement: Supplementary file 2 — Figure S1. Tub activated the AMPK pathway. [file 41419_2020_2915_MOESM2_ESM.png]

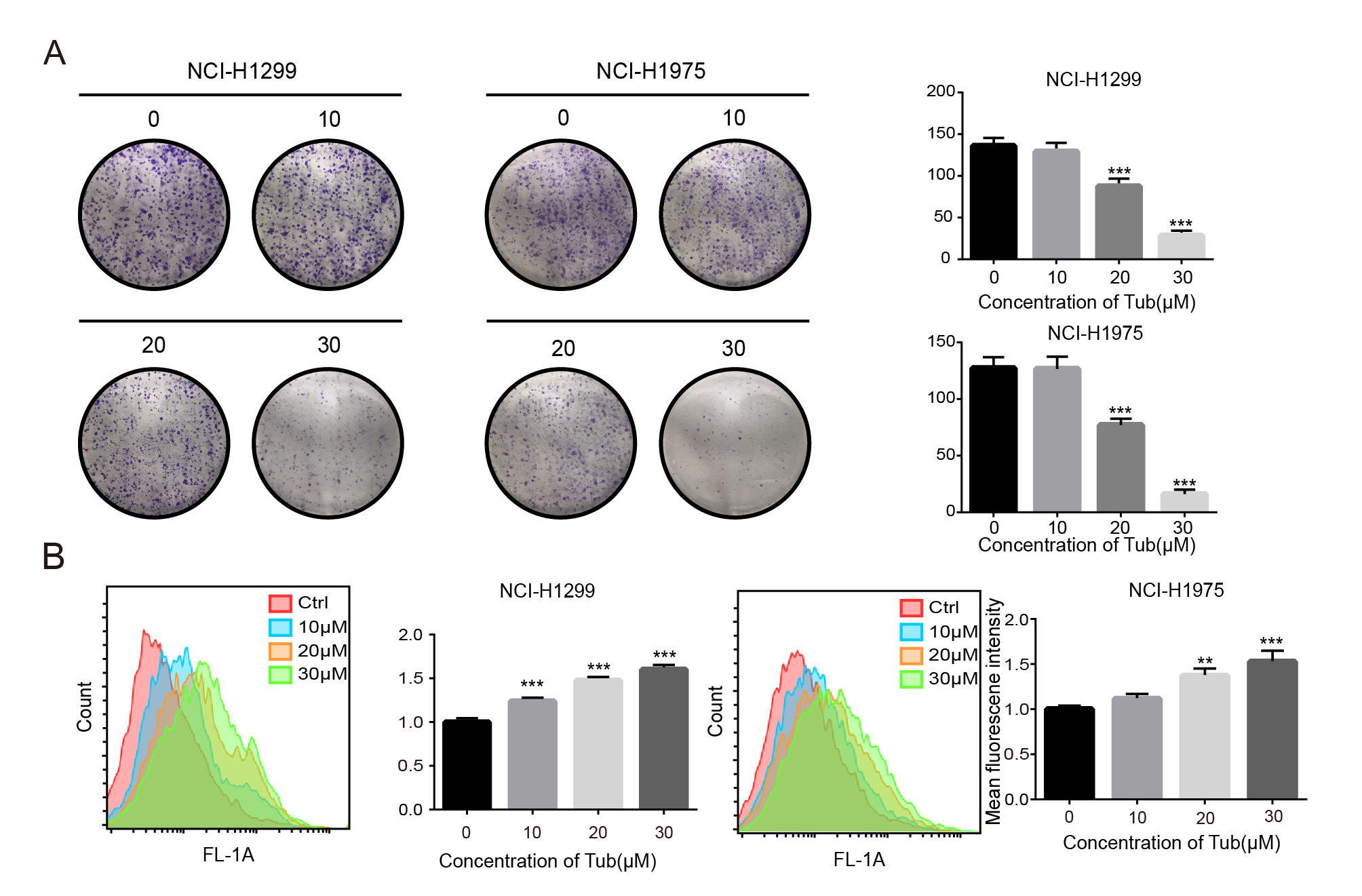

Supplement: Supplementary file 3 — Figure S2. Tub inhibited lung cancer cell proliferation. [file 41419_2020_2915_MOESM3_ESM.png]

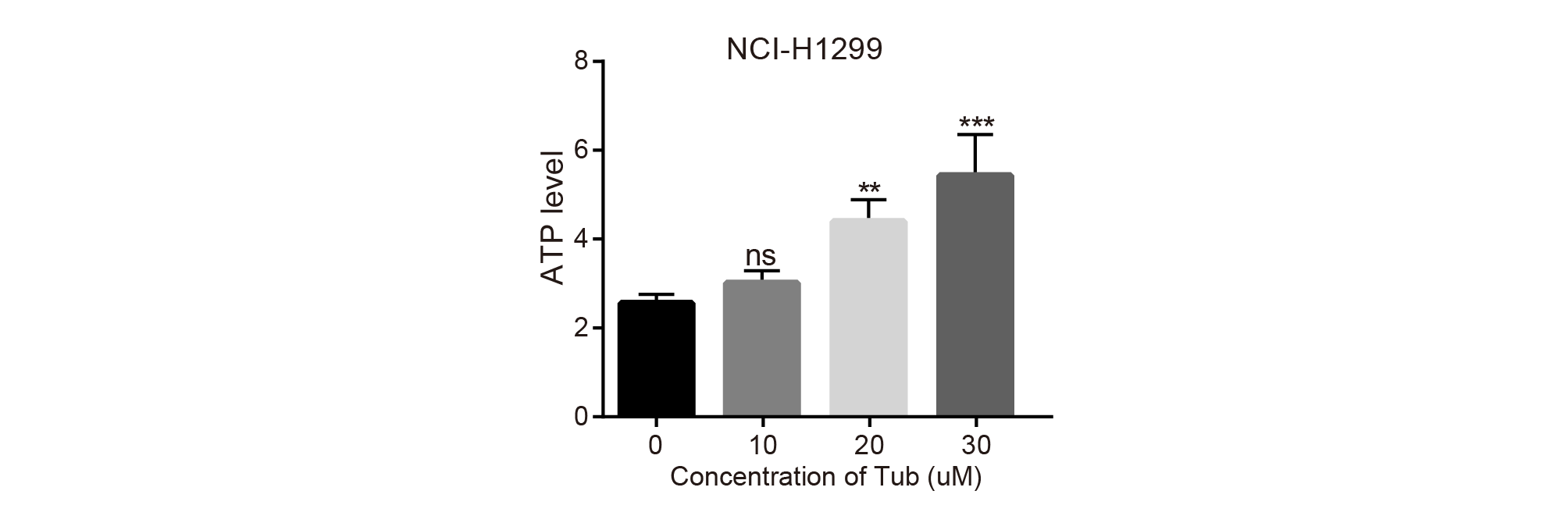

Supplement: Supplementary file 4 — Figure S3. Tub did not decrease the ATP level in NCI-H1299 cells. [file 41419_2020_2915_MOESM4_ESM.png]

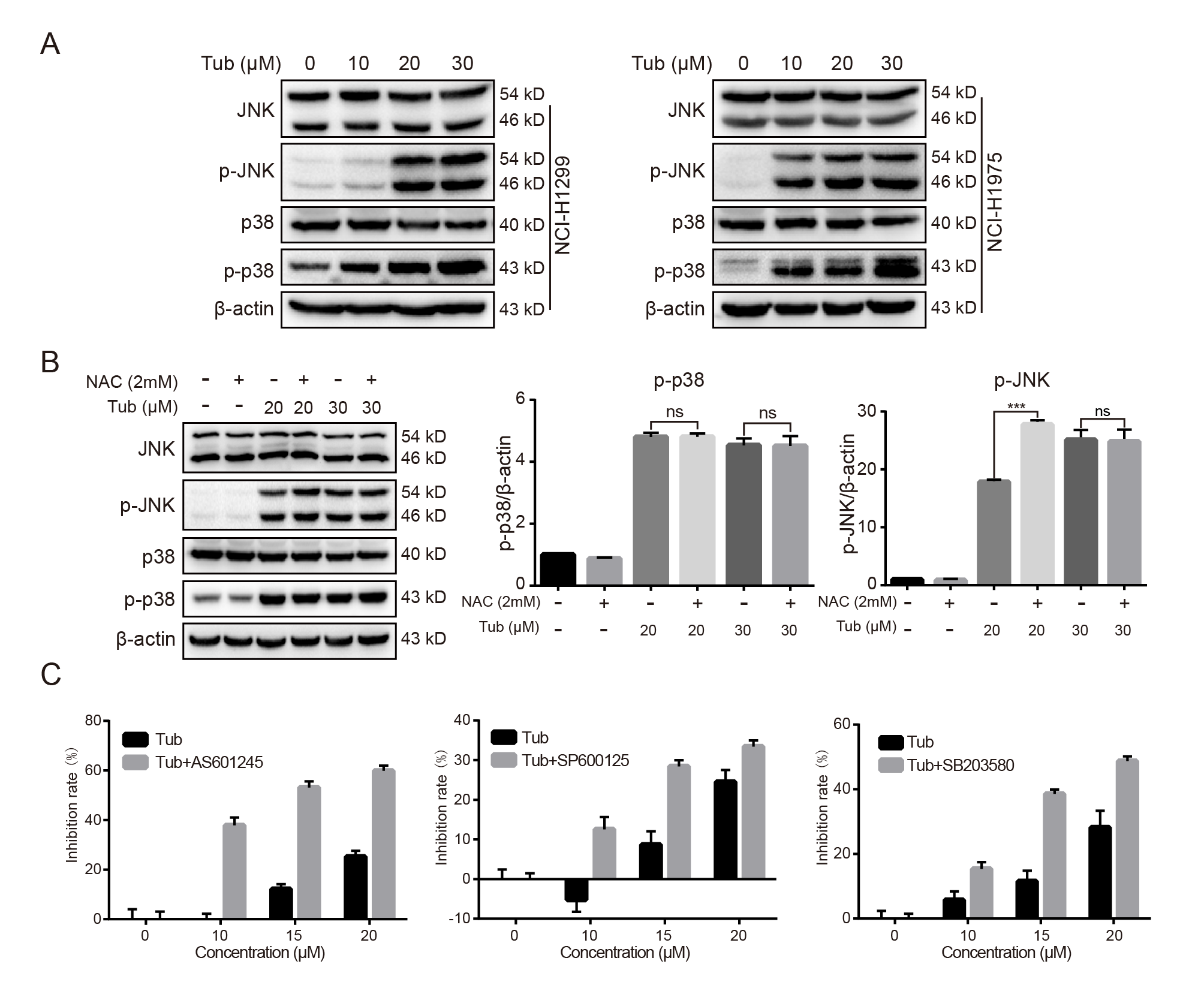

Supplement: Supplementary file 5 — Figure S4. Tub activated the MAPK pathway, but this pathway was not associated with Tub-induced lung cancer cell inhibition. [file 41419_2020_2915_MOESM5_ESM.png]
